# Decorin-Mediated Inhibition of Human Trophoblast Cells Proliferation, Migration, and Invasion and Promotion of Apoptosis *In Vitro*

**DOI:** 10.1155/2015/201629

**Published:** 2015-08-19

**Authors:** Yanfen Zou, Xiang Yu, Jing Lu, Ziyan Jiang, Qing Zuo, Mingsong Fan, Shiyun Huang, Lizhou Sun

**Affiliations:** ^1^Department of Obstetrics and Gynecology, The First Affiliated Hospital of Nanjing Medical University, 300 Guangzhou Road, Nanjing 210029, China; ^2^Department of General Surgery, The Second Affiliated Hospital of Nanjing Medical University, 121 Jiangjiayuan, Xiaguan District, Nanjing 210000, China

## Abstract

Preeclampsia (PE) is a unique complication of pregnancy, the pathogenesis of which has been generally accepted to be associated with the dysfunctions of extravillous trophoblast (EVT) including proliferation, apoptosis, and migration and invasion. Decorin (DCN) has been proved to be a decidua-derived TGF-binding proteoglycan, which negatively regulates proliferation, migration, and invasiveness of human extravillous trophoblast cells. In this study, we identified a higher expression level of decorin in severe PE placentas by both real-time reverse transcription-polymerase chain reaction (qRT-PCR) and immunohistochemistry (IHC). And an inhibitory effect of decorin on proliferation, migration, and invasion and an enhanced effect on apoptosis in trophoblast cells HTR-8/SVneo and JEG-3 were validated *in vitro*. Also the modulations of decorin on trophoblast cells' metastasis and invasion functions were detected through regulating the matrix metalloproteinases (MMP2 and MMP9). Thus, we suggested that the contribution of decorin to the modulation of trophoblast cells might have implications for the pathogenesis of preeclampsia.

## 1. Introduction

Preeclampsia (PE) has been proved to be a crucial cause for the increased maternal and perinatal mortality and morbidity, with a worldwide considerable incidence of 2–8% [1]. It is known as a pregnancy-specific disease with a new occurrence of hypertension and proteinuria during the second stage of pregnancy as its main clinical characteristic. The release of the symptoms happens only along with the delivery of the baby and placentas [2]. Since the enigmatic prognosis of this disease, more and more research has been continuously done to overcome its adverse outcomes.

As for the pathogenesis of PE, the hypotheses including inflammatory cytokines theory [3–5], insufficient remodeling of the maternal spiral artery [6–8], dysfunctional oxidative stress [9, 10], and genetic and dietary reasons [11] are involved. The dysfunctional state of oxidative stress is reported to activate a series of apoptotic signaling pathways which used to keep balance under normal circumstances [12]. And the aberrant villous trophoblast apoptosis has been discovered to be associated with the pathogenesis of PE [13]. Also the impaired remodeling of the maternal spiral artery contributes importantly to PE development, which strongly relies on the decreased migration and invasion potential of placental extravillous trophoblast (EVT) cells [14]. In normal conditions, the EVT cells migrate to the endovascular of the artery in order to invade and replace it, which is known as the endovascular transformation process. Then large diameter, low resistance vessels which could provide steady perfusion to the placenta and baby appear as the consequence of this process [15, 16]. Thus we postulate that the increase of trophoblast cells apoptosis and decrease of migration and invasion ability were closely related to the poor placental implantation and abnormal spiral artery remodeling in pregnancy.

Decorin, primarily synthesized by fibroblasts and myofibroblasts, is a member of the small leucine-rich proteoglycan (SLRP) family [17, 18]. Borbely et al. indicated that decorin was implicated in the invasive activity of EVT cells in pathology of both healthy and disordered placentas [19]. It has been reported that decorin might contribute to the regulation of trophoblast cells' migration and invasion potentials in the mammal placentas [20, 21]. Also, decorin is known to be a functional component of the extracellular matrix with biological functions such as regulating collagen fibrillogenesis and controlling cell proliferation by upregulating p21 [22, 23]. It binds to collagens types I, II, and IV* in vivo* and promotes the formation of fibers with increased stability and changes in solubility [24, 25]. Therefore decorin may contribute to the production of fibers during the remodeling of spiral arteries. However, the detailed influence of decorin on trophoblast cells functions and its involvement in the pathogenesis of PE remain deeply explored.

So in this study, to pursue the effect of decorin gene on trophoblast cells biological functions during PE, we overexpressed decorin gene in trophoblast cells HTR-8/SVneo and JEG-3 cells to identify the role of decorin-mediated cell growth, migration and invasion, and apoptosis* in vitro*.

## 2. Materials and Methods

### 2.1. Patients and Clinical Samples Collection

A group of primipara women aged 20–36 years who were hospitalized for cesarean delivery during December 2011 to March 2012 in our hospital (The First Affiliated Hospital of Nanjing Medical University) was selected for the placenta samples collection. The tissues were washed with sterile phosphate-buffered saline immediately after delivery from the maternal uterus and then kept in the liquid nitrogen until RNA extraction. We obtained the signed version of informed consents from all the women. All trials were approved by the ethics board of our hospital complied with the principles of Declaration of Helsinki guidelines.

### 2.2. Immunohistochemistry Staining

We used immunohistochemistry (IHC) to semiquantitatively and qualitatively detect the decorin protein expression according to the standard method. Briefly, sections of placenta tissues were incubated in 0.3% H_2_O_2_-methanol for 20 min to block endogenous peroxidase activity after being deparaffinized and dehydrated by xylene and rehydrated by 100% alcohol. Then we irritated the sections in 0.1 M citrate buffer in a microwave oven for antigen retrieval and incubated them with 10% bovine serum albumin to block nonspecific antibody binding. The primary antibody (rabbit anti-DCN; 1 : 500, Santa Cruz Biotechnology) and peroxidase-conjugated secondary antibody (1 : 1,000; Beijing ZhongShan Biotechnology CO., Beijing) were utilized to incubate the sections in turn. Finally, a digital photomicrograph was applied to capture photos of stained sections.

### 2.3. Cell Culture and Treatment

One of the cell lines used in this study is HTR-8/SVneo [26], which is derived from a short-lived primary EVT and was kindly provided by Dr. Charles Graham, Queen's University, Canada. With the similarity, it has been adopted to simulate trophoblast cells in a number of researches [27, 28]. It was cultured in an incubator with temperature of 37°C and 5% humidified CO_2_ perfusion. The medium is RPMI1640 which is supplemented with 10% heat-inactivated fetal bovine serum (FBS), 100 U/mL penicillin, and 100 *μ*g/mL streptomycin.

The other cell line choriocarcinoma cell JEG-3 (bought from Cell Bank of Chinese Academy of Sciences in Shanghai) was maintained in DMEM medium, with the same condition mentioned above. Both cells were transiently transfected with overexpression plasmids of decorin (pEGFP-DCN) and an empty vector (pEGFP-N1) as control. The overexpression plasmid of decorin was conducted by Invitrogen Inc. After transfection for 48 hours, we collected the cells to test the transfection efficiency by observing the fluorescence efficiency under a fluorescence microscopy and overexpression efficiency via quantitative real-time PCR (qRT-PCR), respectively.

### 2.4. Cell Proliferation Assays

We used a MTT kit (Sigma) and colony formation assay to analyze the viability of both cells. According to the MTT instructions, an enzyme-linked immunosorbent assay plate reader was used to measure the absorbance of treated cells at 490 nm. As to the colony formation assay, we sowed 500 cells into each 6-well plate with 10% FBS medium for 2 weeks. After being fixed with methanol, the cell colonies were stained with 0.1% crystal violet (Sigma) for 15 minutes. The number of stained colonies was counted under an inverter microscope. Random experiments were done more than 3 times.

### 2.5. *In Vitro* Cell Migration and Invasion Assays

After being transfected as mentioned previously for 24 hours, 5 × 10^5^ cells were resuspended in 1% FBS medium and placed into the upper well of a transwell chamber (Millipore, Billerica, MA), while 10% FBS medium was added into the lower well as a chemoattractant. The diameter of the membrane pore of the transwell chamber is 8 *μ*m. There exists little difference between migration and invasion assays in the following steps. For invasion assay, the upper chamber was coated with 100 *μ*L growth factor reduced matrigel (BD Biosciences, Oxford, UK) and allowed to set at 37°C for at least 30 min in advance, while the migration asssay was not coated. After another 24-hour culture, the number of cells that migrated to the lower surface was fixed by crystal violet and examined.

### 2.6. Western Blotting (WB) Analysis

After treatment, cells were lysed by using RIPA protein extraction reagent (Beyotime) which is supplemented with a protease inhibitor cocktail (Roche) and phenylmethylsulfonyl-fluoride (Roche). The concentration of proteins in each sample was tested by a Bio-Rad protein assay kit. The protein extractions (50–100 *μ*g) were separated by polyacrylamide gel electrophoresis containing 10% sodium dodecyl sulfate and then transferred to polyvinylidene difluoride membranes or 0.22 mm nitrocellulose (Sigma). Then we used specific antibodies in a concentration of 1 : 1000 (DCN, Santa Cruz; Caspase-3, Bcl-2, MMP2, and MMP9, Cell Signaling Technology) to incubate them.

The horseradish peroxidase-conjugated goat anti-rabbit IgG or goat anti-mouse IgG (1 : 1,000; Beijing ZhongShan Biotechnology CO., Beijing) was adopted as the secondary antibody. In order to visualize the bands, the ECL chromogenic substrate was used. The intensity of the bands was quantified by Quantity One software (Bio-Rad). Glyceraldehyde-3-phosphate dehydrogenase (GAPDH, 1 : 1000, Santa Cruz) was applied as control.

### 2.7. RNA Extraction and Real-Time RT-PCR

Trizol reagent (Invitrogen Life Technologies) was utilized for total RNA extraction from cells and placenta tissues. The RNA (1 mg) was reversed to cDNA by using a Reverse Transcription Kit (Takara). The qRT-PCR with the use of An ABI 7500 and the reagent of SYBR Premix Ex Taq (TaKaRa) was to determine the expression levels of amplification product. We applied GAPDH as internal control. The sequences of the primers are as follows: DCN (Forward: 5′-CGAGTGGTCCAGTGTTCTGA-3′, Reverse: 5′-AAAGCCCCATTTTCAATTCC-3′); GAPDH (Forward: 5′-GACTCATGACCACAGTCCATGC-3′, Reverse: 5′-AGAGGCAGGGATGATGTTCTG-3′).

### 2.8. Flow Cytometry (FCM)

After transfection the HTR-8/SVneo and JEG-3 cells were harvested to analyze the apoptosis by flow cytometry (FACScan; BD Biosciences) equipped with CellQuest software (BD Biosciences). The annexin V-APC and 7-amino-actinomycin (7-AAD) (BD Biosciences) were used to label the cells. The number of cells including living, necrotic, early apoptotic, and lately apoptotic cells was counted. And the early and lately apoptotic cells were chosen for further comparison.

As to cell-cycle analysis, cells after treatment were stained with propidium oxide by the Cycle Test Plus DNA Reagent Kit (BD Biosciences) according to the protocol and then analyzed by FACScan (BD Biosciences). The cells were sorted into G0-G1, S, and G2-M phase and the percentages of each phase were counted and compared. These assays were repeated more than three times.

### 2.9. Statistical Analysis

A SPSS 17.0 statistical software package (SPSS Inc., Chicago, IL, USA) was used for statistical analysis. The patients' clinical data analysis was processed by One-Way ANOVA, and as to the cells experiments, paired samples *t*-test was used. The data appeared as mean ± SD (standard deviation, SD). *P* values of less than 0.05 were considered statistically significant.

## 3. Results

### 3.1. Clinical Characteristics and Expression Level of Decorin in Human Placenta and Normal Tissues

**Figure 1:**
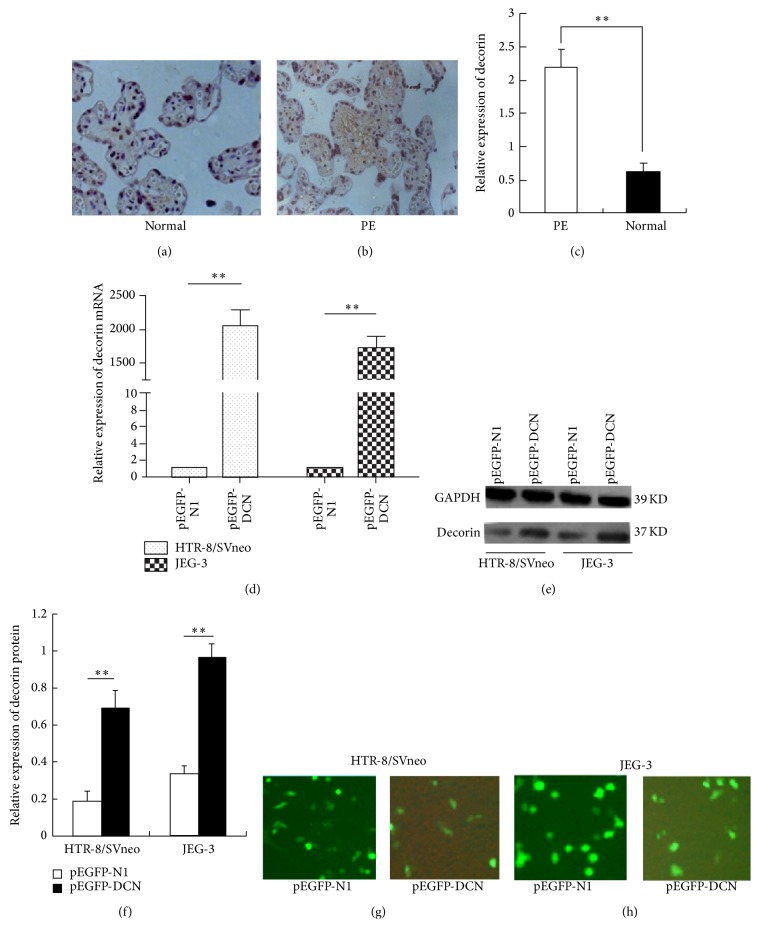
Expression of decorin in preeclampsia placentas compared with normal and decorin-overexpression efficiency in trophoblast cells. (a) Relative expression of decorin was 71.8% higher in preeclampsia placenta tissues compared to the normal pregnancies, as determined by qRT-PCR. (b) The mRNA expression of decorin in HTR-8/SVneo and JEG-3 cells transfected with pEGFP-DCN, detected by qRT-PCR. ((c) and (d)) The protein expression of decorin in HTR-8/SVneo and JEG-3 cells transfected with pEGFP-DCN, detected by Western blotting. Values are represented as mean ± SEM (^∗∗^ *P* < 0.01).

The expression level of decorin was detected in 9 PE and 12 normal placenta tissues by using immunohistochemical staining. The results showed that decorin protein was greatly upregulated in PE but was expressed at lower level in normal placenta tissues (Figures 1(a) and 1(b)). Also, the qRT-PCR analysis was conducted by comparing 30 PE placentas to 30 normal pregnant ones. The expression level of decorin mRNA was also significantly higher in PE placentas than that of the normal ones (Figure 1(c)).  Table 1 shows the patients' clinical characteristics in detail.

### 3.2. Exogenous Overexpression of Decorin in HTR-8/SVneo and JEG-3 Cells

The HTR-8/SVneo and JEG-3 cells that were sowed into 6-well plates previously were transfected with overexpression plasmids targeting decorin. The overexpression efficiency was detected by both qPCR (Figure 1(d), *P* < 0.01) and Western blotting assay (Figures 1(e) and 1(f), *P* < 0.01) in both cells after being transfected with pEGFP-DCN and empty vector for 48 h. The qPCR presented a 2091-fold and 1708-fold overexpression of decorin by pEGFP-DCN and WB analysis showed a 15% and 43% upregulation of decorin as compared to control in HTR-8/SVneo and JEG-3 cells, respectively. Of course, the transfection efficiency was detected by observing the fluorescence efficiency (more than 75%) under a fluorescence microscopy in both cells (Figures 1(g) and 1(h), *P* < 0.05). These results indicated that the overexpression of decorin was effectively in our study.

### 3.3. Modulation of Decorin Expression in Cell Migration and Invasion* In Vitro*

**Figure 2:**
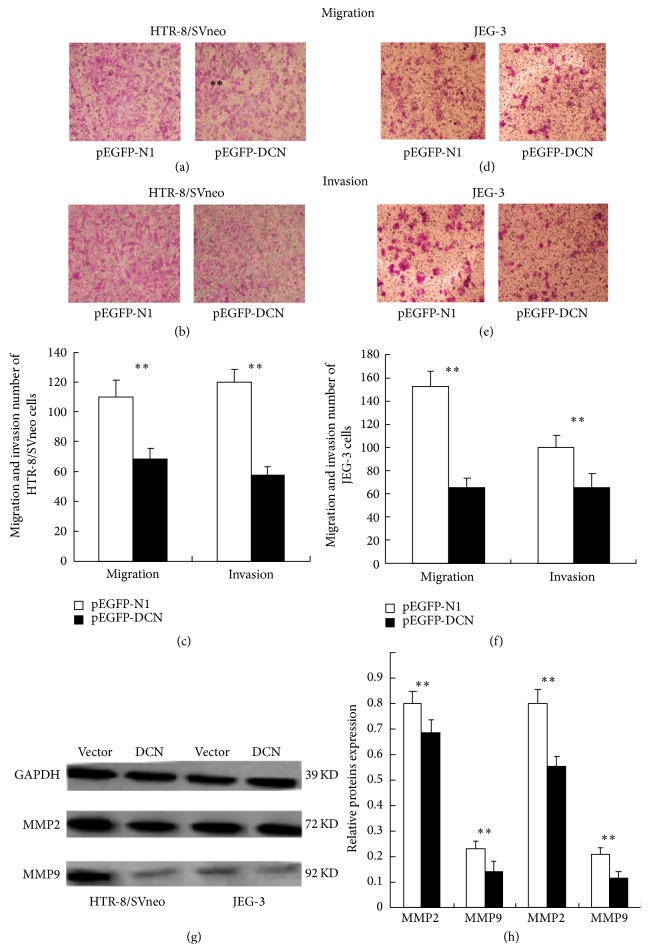
The migration and invasion capacity of trophoblast cells transfected with pEGFP-DCN and control. ((a) and (b)) HTR-8/SVneo cells treated with decorin overexpression presented significantly inhibited migration (a) and invasion (b) potentials compared to control. (c) The histogram showed the statistical data of (a) and (b). (d) The migration ability of JEG-3 cells treated with decorin overexpression was significantly lower than that of the control, as determined by transwell assays. (Values are mean ± SEM; ^∗^ *P* < 0.05; ^∗∗^ *P* < 0.01.) (e) The invasion ability of JEG-3 cells treated with decorin overexpression was significantly lower than control. (f) The histogram showed the statistical data of (e) and (f). ((g) and (h)) Western blotting analysis of MMP2 and MMP9 protein in pEGFP-DCN or empty vector transfected HTR-8/SVneo and JEG-3 cells. (Values are mean ± SEM; ^∗^ *P* < 0.05; ^∗∗^ *P* < 0.01.)

It was determined by transwell assays that the migratory and invasive capacity of cells transfected with pEGFP-DCN reduced by approximately 37.6% and 51.88% of HTR-8/SVneo cells (Figures 2(a), 2(b), and 2(c), *P* < 0.01), respectively, and 57.3% and 34.8% of JEG-3 cells (Figures 2(d), 2(e), and 2(f), *P* < 0.01), respectively, as compared to that of control. Moreover, matrix metalloproteinases, MMP2 and MMP9, also presented a decrease under the influence of decorin overexpression in both cells (Figures 2(g) and 2(h), *P* < 0.01). Thus, these data proved that decorin might be closely associated with the inhibition of migration and invasion behaviors in trophoblast cells.

### 3.4. Overexpression of Decorin Inhibited Cell Growth and Proliferation and Promoted Cell Apoptosis* In Vitro*

**Figure 3:**
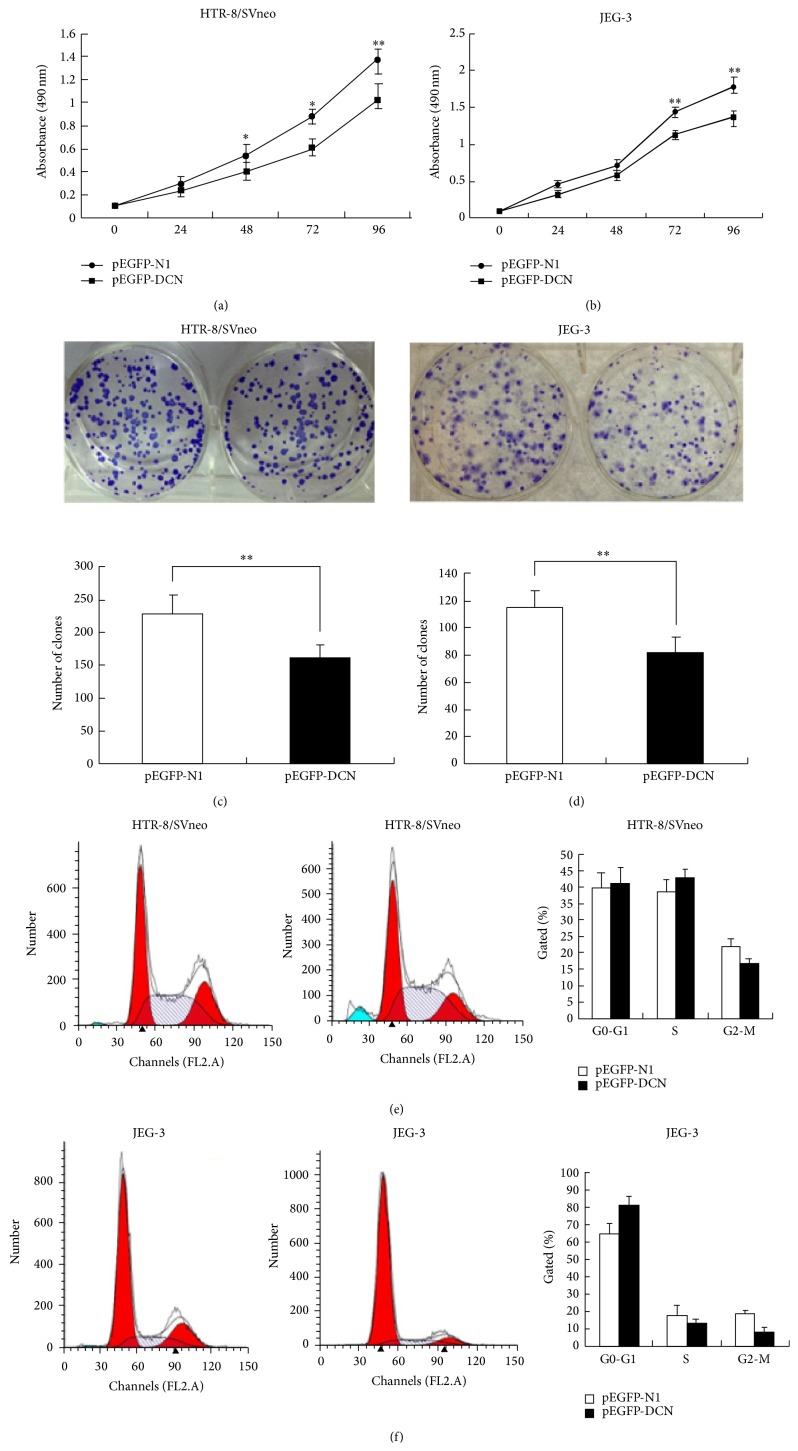
Effects of decorin on growth and proliferation of trophoblast cells. ((a) and (b)) The proliferation ability of HTR-8/SVneo and JEG-3 cells was inhibited in pEGFP-DCN group compared to control, as identified by MTT assays. ((c) and (d)) Colony-forming assay showed a decrease of cells proliferation in pEGFP-DCN group compared to empty vector both in HTR-8/SVneo and in JEG-3 cells. ((e) and (f)) Cell-cycle analysis was performed 48 h following the treatment of HTR-8/SVneo and JEG-3 cells with pEGFP-DCN or empty vector. The DNA content was quantified by flow cytometric analysis. Values are represented as mean ± SEM (^∗∗^ *P* < 0.01).

The significant increase of decorin expression in PE placenta samples prompted us to explore the possible biologic significance of decorin in the pathogenesis of PE. To determine whether decorin affects trophoblast cells growth, we conducted MTT assay to detect cell growth viability in pEGFP-DCN transfected HTR-8/SVneo and JEG-3 cells compared to that of control (Figures 3(a) and 3(b), *P* < 0.01). Also, the impact of decorin on cell proliferation was assessed by colony formation assay. According to the colony formation assay, we found that cells transiently transfected with pEGFP-DCN had significantly reduced proliferation of cells compared with that of cells transfected with pEGFP-N1 (Figures 3(c) and 3(d), *P* < 0.01). Additionally, flow cytometric analysis was used to further examine whether the inhibition of decorin on cell proliferation reflected cell-cycle arrest. The cell-cycle analysis showed that cells transfected with pEGFP-DCN had an obvious cell-cycle arrest at the G1-G0 phase with a decreased G2–S-phase compared to that of negative control (Figures 3(e) and 3(f), *P* < 0.01).

**Figure 4:**
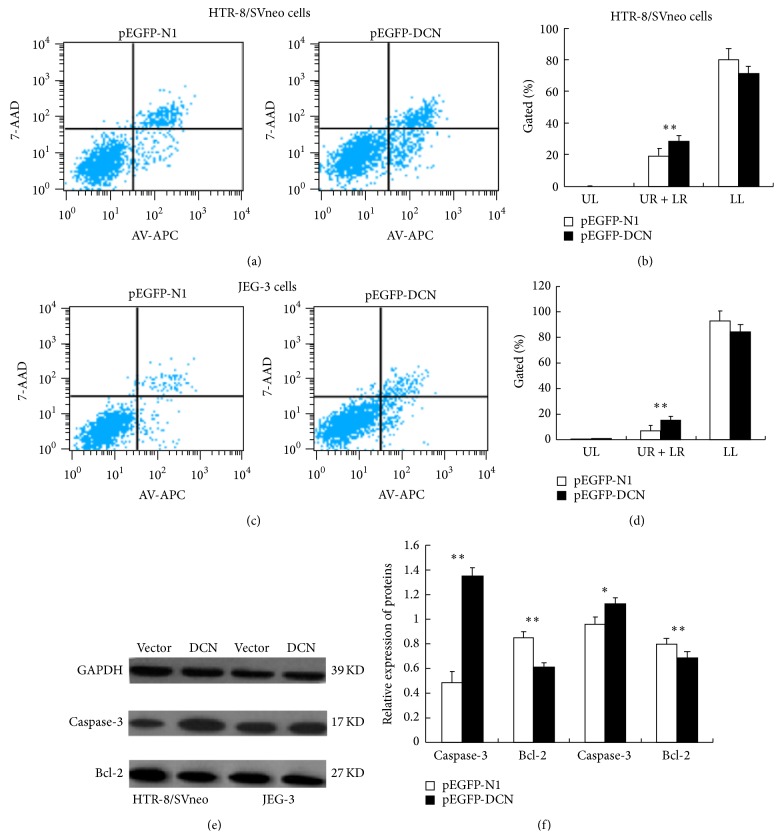
Cell apoptosis detection by flow cytometry and Western blotting assays. ((a) and (b)) HTR-8/SVneo cells transfected with pEGFP-DCN showed a significantly higher rate of apoptosis by flow cytometry. ((c) and (d)) JEG-3 cells transfected with plasmid overexpressing decorin showed a significant increase in apoptotic rate as compared to that of the empty vector as demonstrated by flow cytometry. ((e) and (f)) Western blotting analysis of apoptotic protein in cells transfected with pEGFP-DCN displayed an increase of cleaved Caspase-3 (17KD), while it displayed a reduction of Bcl-2 (27KD). (Values are mean ± SEM; ^∗^ *P* < 0.05; ^∗∗^ *P* < 0.01.)

Furthermore, in order to evaluate whether the trophoblast cells growth and proliferation potential was affected by cell apoptosis, we performed flow cytometry to detect the apoptotic cells and Western blotting assays to identify apoptotic proteins in both cells treated with pEGFP-DCN. When HTR-8/SVneo and JEG-3 cells were transfected with pEGFP-DCN, a significant increase of apoptosis was observed as compared to control (Figures 4(a), 4(b), 4(c), and 4(d), *P* < 0.01). And the apoptotic protein cleaved Caspase-3 was signifiantly increased in cells transfected with pEGFP-DCN while the antiapoptotic protein Bcl-2 decreased (Figures 4(e) and 4(f), *P* < 0.01). These results indicated that enhanced decorin expression could repress trophoblast cells growth and proliferation and promote cells apoptosis.

## 4. Discussion

There have been more and more reports that evidenced decorin's inhibitory effects on tumorigenesis and overexpressed decorin could inhibit cancer cells growth and metastasis and promote apoptosis [29–32], while fewer reports [21] existed referring to its role in trophoblast cells' functions, even pathogenesis of PE. Herein, we verified by qRT-PCR that decorin mRNA was markedly increased in PE placentas than in normal pregnancy. And a positive relationship between the expression of decorin proteins detected by IHC and PE suggests that decorin may be involved in pathogenesis of PE. Thus a series of studies about the role of decorin in the biological functions of trophoblast cells involved in PE pathogenesis were conducted by us. These results may not be consistent with the results by Chui et al. who reported a reduced expression of decorin in 21 cases of PE compared to normal [33]. We thought this may be attributed to the fewer samples cases and ethnic differences. However, the effect of decorin on the biological functions of EVT cells is consistent with other researchers [20].

The* in vitro* data in our study proved that the growth and proliferation of the HTR-8/SVneo cells were decreased and their apoptosis rate was increased when decorin was upregulated in trophoblast cells HTR-8/SVneo and JEG-3. These assays evidenced that decorin could modulate the biological activity of trophoblast cells by inhibiting cells growth. The mechanism of cell growth modulation by decorin may be through interaction with growth factor receptors at the cell surface. It was reported that decorin could modulate and induce signal transduction along pathways involving the EGFR [34] and the IGFR [35] among others. On the other hand, Guidetti et al. proved that exogenous recombinant decorin or de novo expression of decorin could downregulate the endogenous expression of proangiogenic factor, VEGF, as well as that of fibroblast growth factor-2 (FGF-2) [36] and suppressed the tumorigenicity of human colon carcinoma cells both* in vitro* and* in vivo*. However, the detailed pathways through which decorin regulates trophoblast cells' biological behaviors in PE need to be multidirectionally explored.

Generally, the disorder of maternal spiral arteries remodeling might contribute to the pathogenesis of PE. And our findings in this work indeed indicated that decorin overexpression could inhibit trophoblast cells migration and invasion and promote apoptosis. Decorin, localized to the placental fetal blood vessel walls, is involved in trophoblast cells migration and invasion [20] and endothelial cell development as well. PE is associated with reduced perivascular and endovascular trophoblast cell invasion of the maternal spiral arteries [37] and increased cells apoptosis. These findings indicated that decorin might be one of the crucial factors for the conversion from endometrial epithelial cells to trophoblast cells in remodeling of maternal spiral arteries.

MMPs, a family of zinc-dependent proteolytic enzymes, are expressed in extravillous trophoblasts and involved in the process of trophoblast cells degrading the extracellular matrix and remodeling normal structure [38]. Reduced MMPs levels in the deciduas and placentas were observed in PE women and contribute importantly to the invasion of extravillous trophoblasts into the spiral arteries wall [39]. Thus, the modulation of decorin on trophoblast cells' metastasis and invasion functions may be through interacting with MMP. Then in our study, we found a block of MMP2 and MMP9 proteins after trophoblast cells transfected with decorin-overexpression plasmid. Therefore, the disrupted expression of decorin might cause implications for inadequate conversion of maternal spiral arteries through acting with MMPs, leading to placental abnormalities or PE. However, the detailed modulation mechanism remains unclear.

In conclusion, our study showed that decorin was significantly upregulated in PE placentas and was involved in regulating trophoblast cells biological functions. Decorin might be associated with the pathogenesis of PE and further insights into the deep basis of its function and clinical implications may contribute to the early diagnosis and treatment of PE.

## Figures and Tables

**Table 1:** Clinical characteristics of normal and preeclamptic pregnancies.

Variable	PE (*n* = 30)	*N* (*n* = 30)	*P* ^a^ value Control vs PE
Maternal age	30.2 ± 5.7	30.6 ± 3.5	*P* > 0.05 (0.1388)
Proteinuria (g/day)	6.32 ± 0.85	<0.3	*P* < 0.01 (0.0065)
Gestational age (week)	36.5 ± 3.7	39.1 ± 1.2	*P* > 0.05 (0.0976)
Systolic blood pressure, mm Hg	169 ± 20.1	112 ± 6.8	*P* < 0.01 (0.0037)
Diastolic blood pressure, mm Hg	115 ± 12.8	77 ± 7.1	*P* < 0.01 (0.0094)
Body weight of infant (g)	2582 ± 740	3322 ± 413	*P* < 0.05 (0.0373)
CRP (C-reaction proteins)	8.1 ± 3.1	5.9 ± 2.9	*P* > 0.05 (0.0966)

All results are presented as mean ± SD. SD: standard deviation.

^a^Obtained by 1-way analysis of variance using SPSS 13.0 software (SPSS Inc., Chicago, IL).
